# Ultrasound Imaging Versus Morphopathology in Cardiovascular Diseases: The Heart Failure

**DOI:** 10.1186/1476-7120-5-5

**Published:** 2007-01-30

**Authors:** Giorgio Baroldi, Riccardo Bigi, Lauro Cortigiani

**Affiliations:** 1 Institute of Clinical Physiology, National Research Council, Milan and Pisa, Italy; 2 Cardiology, University School of Medicine and Centro Diagnostico Italiano, Milan, Italy; 3 Cardiovascular Unit, "Campo di Marte" Hospital, Lucca, Italy

## Abstract

This review article summarizes the results of histopathological studies to assess heart failure in humans. Different histopathological features underlying the clinical manifestations of heart failure are reviewed. In addition, the present role of echocardiographic techniques in assessing the failing heart is briefly summarized.

## Review

Any cardiac disease may end in congestive heart failure (CHF), including coronary heart disease. Since mortality has been reduced in acute coronary syndromes, the latter will be the main condition resulting in CHF. This concept stimulated a systematic, quantitative study in 144 hearts with irreversible insufficiency excised at transplantation compared with 144 hearts without CHF both from patients and normal subjects dead from accident [1]. The aim of this review is to discuss a consensus on CHF which, independently of the underlying disease, is considered: "secondary to absolute or relative ischemia, remodelling of heart shape by enlarged volume, increased mass, cardiac wall thinning, lengthening of cardiomyocytes, continuous infarct expansion, scar formation, apoptosis, hypertrophy, slippage of myocardial cells and increased interstitial fibrosis, and neurohormonal activation" [2].

Our 144 patients with irreversible CHF included 63 with chronic coronary heart disease, 63 with dilated cardiomyopathy and 18 with chronic valvular heart disease. All had a similar clinical pattern. The advantage is that surgically excised hearts are free of any change which may occur in the terminal, agonic phase and interfere with other autoptic findings.

The main data supporting this review are synthesize in Table 1 and main items in the consensus to be discussed are:

**Table 1:** Main findings in 144 hearts with and 144 hearts without congestive heart failure

**Source**	**Total**	**Age**	**Heart**			**Myocardial fibrosis**				**Intermyocyte lymphocytic infiltration**				**Catecholamine necrosis**			**Colliquative myocytolysis**			
	**cases**	**yrs**	**weight**	**trans.Ø**	**Ant.LV**	**no**	**20%**		**FI**	**absent**	**-Mn**	**+Mn**	**Foci**	**+**	**Foci**	**Myocells**	**no**	**[% cells]**		
			**g**	**mm**	**mm**		**<**	**>**					**×100 mm**^**2**^					**<25**	**25–50**	**>50**

Ischemic heart	63	51 ± 8	565 ± 108	137 ± 14	12 ± 4	-	11	52	17 ± 8	9	38	17	0.2 ± 0.05	59	1 ± 1	10 ± 14	1	14	39	9
Dilated cardiom.	63	42 ± 12	639 ± 155	148 ± 15	15 ± 4	8	54	1	2 ± 3	8	42	18	0.4 ± 0.18	53	2 ± 6	12 ± 26	3	28	28	4
Valvulopathy	18	46 ± 8	827 ± 179	147 ± 15	16 ± 4	-	14	4	4 ± 4	3	12	7	0.3 ± 0.09	14	1 ± 2	5 ± 6	1	2	14	1
Total+CHF*	**144**	**46 ± 9**	**677 ± 147**	**144 ± 15**	**14 ± 4**	**8**	**79**	**57**	**8 ± 5**	**20**	**92**	**42**	**0.3 ± 0.09**	**126**	**1 ± 3**	**9 ± 15**	**5**	**44**	**81**	**14**
Chagas	34	49 ± 13	464 ± 110	114 ± 18	15 ± 3	9	18	7	7 ± 8	-	34	31	13 ± 2	34	3 ± 6	30 ± 51	28	6	-	-
AIDS	38	31 ± 10	368 ± 67	91 ± 21	13 ± 3	34	4	-	0.04 ± 1	14	16	9	1.5 ± 0.7	25	4 ± 11	13 ± 27	33	3	2	-
Brain hemorrhage	27	58 ± 12	434 ± 91	107 ± 15	18 ± 7	12	15	-	0.4 ± 6	15	7	-	2 ± 1.8	24	26 ± 34	67 ± 104	26	-	1	-
Head trauma normal	45	42 ± 17	364 ± 47	104 ± 15	12 ± 6	45	-	-	-	29	11	-	5 ± 3	9***	10 ± 18	23 ± 31	45	-	-	-
**Total without CHF**	**144**	**45 ± 17**	**408 ± 79**	**104 ± 17**	**15 ± 5**	**100**	**37**	**7**	**2 ± 5**	**58**	**68**	**40**	**5 ± 1.9**	**92**	**11 ± 17**	**33 ± 53**	**132**	**9**	**3**	**-**

* Heart excised at transplantation; ** Mn, myocyte necrosis non [-] or [+] associated with lymphocytes; *** Survival < 1 hour. No necrosis in instantaneous death; Ant LV, thickness anterior left ventricle; trans. Ø, transvers diameter

### 1. Myocardial fibrosis

**Figure 1:**
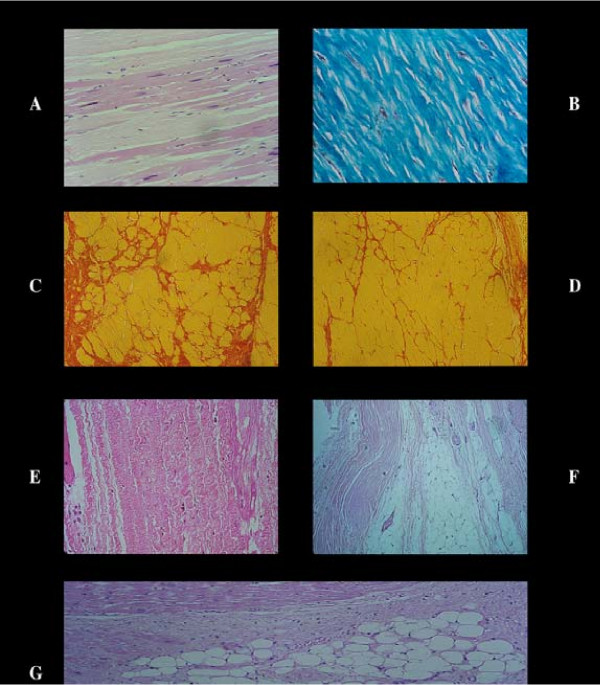
Stretched, i.e. elongation of sarcomeres and nuclei in viable myocardial cells in an old aneurysm of the left ventricular wall with dense fibrosis [A]. Dense and compact fibrosis as end result of a repair process of an infarct. The collagen fibers are straight and closed together [B]. In contrast in hearts with congestive heart failure the myocardial fibrosis is very mild [C, D] showing a corkscrew aspect of the collagen fibers [E]. This means an adaptation of the collagenogenesis to the contraction cycle without any capability to reduce or stop the latter. Furthermore, the fibrous tissue may metaplasize in adipose tissue which substitutes large fibrous area [F, G]. A fact to have in mind when quantifying the size of a scar or measuring by nuclear method the myocardial viability.

This variable assumes a central pathogenic meaning since it results (ischemia → continuous infarct expansion → scar) in fibrous, non contracting myocardium ending in progressive insufficiency. In all the three conditions examined, the extent of myocardial fibrosis (Figure 1) was unable to explain myocardial global insufficiency. Even in coronary heart disease group the "fibrous index" (extent percent of myocardial fibrosis found in 16 histologic slides in each heart) showed that the amount of histologically viable myocardium was 83% (98% in dilated cardiomyopathy, 94% in chronic valvulopathy). Furthermore, the increased interstitial fibrosis has ondulate or wavy collagen fibers (Figure 1). This means that collagen fiber hyperplasia is a secondary adaptation to myocardial hypertrophy without any blocking effect on contraction. A last warning is that myocardial fibrosis, especially in large scar following an infarct necrosis, often is substituted by adipose tissue which can be misinterpreted by clinical imaging concerning myocardial viability [3].

### 2. Lymphocytic infiltration

In dilated cardiomyopathy the etiopathogenetic hypothesis is that a chronic virus myocarditis resulting in myocardial fibrosis explains this pathologic condition. Both myocardial fibrosis, as already mentioned, and lymphocytic infiltrates, as expression of a virus infection, showed a too low frequency and extent. In particular the number of lymphocytic foci was less than controls in all the insufficient hearts, with a number of elements per focus less than 20. Only in "silent" Chagas patients [apparently healthy subjects serum-positive for this disease who died suddenly and unexpectedly] an extensive lymphocytic myocarditis associated with catecholamine necrosis was observed.

### 3. Ischemia

A clinical study has shown that ischemic episodes are not part of CHF [4]. On the other hand, in coronary artery disease + CHF group an acute, silent infarct was detected in five of 63 hearts, transmural in two and subendocardial in three with a histological age between 20–30 days. In controls, one Chagas patient had a ten day old anterior infarct and four AIDS subjects, all with normal coronary arteries had a microfocal, four day old infarct in the anterior papillary muscle. If ischemia is the cause of CHF we should have a higher frequency of infarct necrosis.

### 4. Apoptosis

This genomically programmed cell death, supposed to be triggered by many risk factors, has been recently advocated to explain different cardiac pathologies in which apoptosis has been indirectly "demonstrated" by TUNEL techniques [5]. This necrosis is defined as shrinkage of single cell with fragmentation of its nucleus forming "apoptotic bodies" easy to recognize within or outside the dead cell. The apoptotic cell then disappears without evidence of collagen substitution [6], a finding never observed in our human and experimental material [5]. The claim, that lost of myocardial cells by apoptosis may explain cardiac insufficiency, is contradicted by the heavy weight of insufficient hearts. Lost of myocytes should correspond to normal, if not subnormal heart weight.

### 5. Thinning cardiac wall/lengthening of myocytes

**Figure 2:**
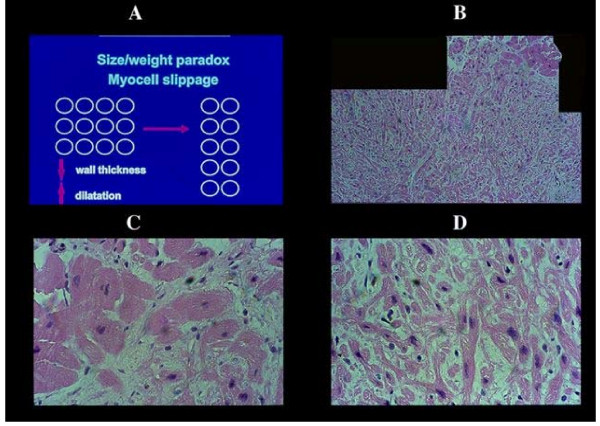
Weight/size paradox in congestive heart failure. The slippage of myocardial cell to explain a normal wall and myocellular size despite a heavy cardiac weight [A], does not consider the myofibrillar bridging between myocells and fibrillar collagen connections. Slippage of myocells, capable to reduce for instance a 3 cm cardiac wall in 1.5 one, should imply an extensive destruction of all interstitial structures [vessels, including lymphatics, and nerves] and consequent widespread tissue damage never seen in this condition. Even a neogenesis of the myocardial cell was never observed. Only once in an endomyocardial biopsy at a previous site of sampling in a transplanted heart, we had the opportunity to see new myocells forming a focus of small elements assuming the aspect of atrio-ventricular node [B, C]. Just to emphasize that a neogenesis when exists can be seen, apparently without integration in the functioning myocardium.

The weight/size paradox i.e. heavy heart weight with both cardiac wall and myocyte normal thickness is explained by dilatation of heart chambers with lengthening by distension of myocardial cells resulting in normal wall thickness. Such a stretch of myocytes does not occur [1] as shown histologically and ultrastructurally (note in Figure 2). Furthermore, congestive heart failure may occur without or with mild chamber enlargement, ventricular dilatation being not an independent prognostic predictor [7].

### 6. Hypertrophy

By cardiac hypertrophy we mean a tridimensional augmentation in number of myofibrils and other components of myocardial cells to compensate an increase of heart work. The myocyte hypertrophy results in a heavy cardiac weight. When insufficiency begins the weight/size paradox can not be explained by longitudinal division of myocytes never demonstrated or by neogenesis of myocytes [8-10], a concept difficult to be accepted since the hypertrophying stimulus persists and the new elements should hypertrophy.

### 7. Slippage of myocytes

To explain the paradox, the other well granted hypothesis is that an interpenetration of myocytes may reduce the cardiac wall thickness. First, such never proved interpenetration may explain the latter and dilatation of heart cavities (Figure 2). However, it does not explain normal thickness of myocytes and implies that there is a destruction of a) connecting myobridges between adjacent myocytes b) their interconnection by collagen matrix, and c) a disruption of all interstitial components as vessels (arterial, terminal bed-capillaries, venous and lymphatics) and nerves never seen in this condition.

### 8. Neurohormonal activation

This assumption has a confirmation in the high frequency of catecholamine necrosis [7] even if the extent of the lesion is very small (Table 1).

The first conclusion is that the pooling of all items of the quoted consensus has no support from morphopathology, but neurohormonal activation which, per se, does not explain congestive failure rather an adrenergic stress prevented by beta-blocking therapy. However, the following two findings not mentioned in this consensus are the following.

### 9. Colliquative myocytolysis

**Figure 3:**
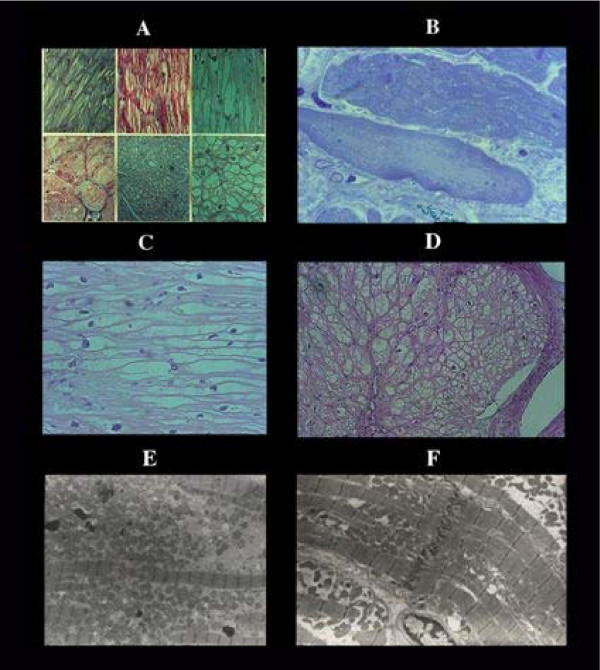
Colliquative myocytolysis. Progressive disappearance of myofibrils [A], with intramyocardial edema [B] resulting in empty sarcolemmal tube seen in longitudinal [C] and transverse section [D]. Note the absence of any type of reaction. E, ultrastructural view of an edematous myocell filled by mitochondria and few contracted myofibrils in contrast with a normal contracted myocell [F] of the same case with congestive heart failure.

This non ischemic lesion [7] is typical of congestive heart failure (Figure 3) and, when other conditions (storage diseases) can be excluded, is a reliable histologic hallmark consequent to myocyte insufficiency with increasing lost of myofibrils, intracellular edema in absence of any type of reaction. Its secondary nature is proved by a lack of relationship with clinical indexes of CHF.

### 10. Endomioelastofibrosis

**Figure 4:**
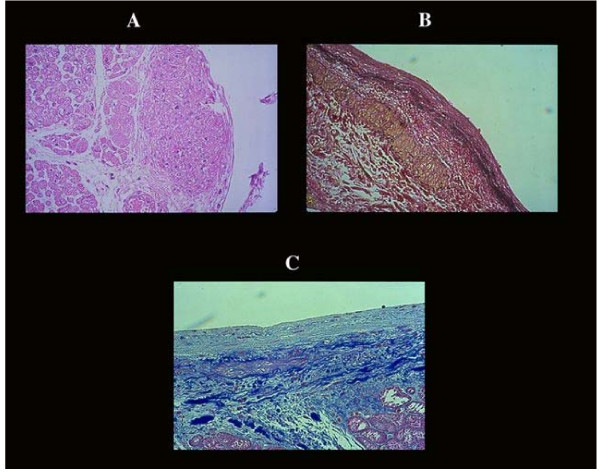
Endomyoelastofibrosis of the endocardium. Thickening of the endocardium is considered consequent to intracavitary thrombi organization. The latter is a relatively rare event. The majority of endocardial thickening is due to the formation of smooth muscle cell bundles [A] with hyperelastosis [B] ending in fibrous replacement [C]. A process similar to that shown in the early pathogenesis of an atherosclerotic plaque, to keep in mind in relation to antithrombotic therapy.

This change is worthy of mention to avoid any confusion with embologenic endocardial thrombosis. The presence of the latter was 21% in coronary heart disease, 10% in dilated cardiomyopathy and 6% of valvulopathic patients with CHF. Most of these endocardial thrombi were organized. Myoelastofibrosis was demonstrated in 98% of congestive hearts being present also in controls (Table 2). It starts as smooth muscle cell nodular proliferation below the normal endothelial cells of the endocardium (Figure 4), followed by elastic fiber hyperplasia and ending in fibrous thickening without evidence of overimposed thrombosis or repair process.

**Table 2:** Frequency of endocardial myoelastofibrosis in different conditions

**Source**			**Endocardial myofibroelastosis**				
	**Cases**	**Absent**	**Nodular myohyperplasia**	**Myofibro elastosis**	**LV**	**RV**	**IV**
Congestive heart failure*	144	3	15	126	129	29	125
Sudden/unexpected coronary death	25	13	2	10	6	6	9
Sudden/unexpected death silent Chagas	34	15	1	18	17	6	10
Transplanted hearts	46	29	6	11	11	5	6
Intracranial brain hemorrhage	27	10	3	14	13	4	9
AIDS	38	16	7	15	11	5	11
Cocaine abusers**	26	19	5	2	2	-	-
Carbon monoxide intoxication**	26	26	-	-	-	-	-
Head trauma**	45	45	-	-	-	-	-
Electrocution**	21	21	-	-	-	-	-

* Hearts excised at transplantation ** Only anterior left ventricle available for study; LV, left ventricle; RV right ventricle; IV, interventricular septum

## Target of Ultrasound Diagnosis: Present and Future

Though various imaging techniques have been introduced for assessing left ventricular function, echocardiography still represents the main choice in the clinical arena due to its unique combination of safety, feasibility, and reliability (Table 3). Both diastolic and systolic function of the heart can be easily explored by echocardiography.

**Table 3:** Noninvasive technique for assessing left ventricular function.

	***Echocardiography***	***Nuclear scan***	***Cardiac CT***	***Cardiac MRI***
Radiation exposure	-	+	+	-
Patient dependence	+	-	-	-
Operator dependence	+	-	-	-
Imaging time	+	++	+	+++
Installation costs	+	++	+++	++++
Operation costs	+	++	+++	++++
Spatial resolution	+++	+	+++	+++
Temporal resolution	+++	+	++	±

CT: computed tomography

MRI: magnetic resonance imaging

Reproduced from Picano E.: Stress Echocardiography. 4th edition. Verlag: Springer; 2003

### 1. Diastolic function

Doppler echocardiography traditionally allows diastolic abnormalities affecting overall cardiac function to be easily detected on routine examination. Inversion of the E/A ratio and prolongation of deceleration time on the transmitral flow pattern represent the first abnormalities which are encountered in the minor stage of diastolic dysfunction. A restrictive filling pattern, reflecting an increased pulmonary capillary pressure, is observed in case of more severe dysfunction and is associated to adverse outcome [11]. More recently, Tissue Doppler Imaging (TDI) has provided robust, relatively unambiguous, well validated tool that is more reliable than the use of pulmonary vein Doppler, flow propagation velocity or load altering techniques [12].

The clinical role of echocardiography for evaluating diastolic function is quite different depending on whether global left ventricular (LV) systolic function is preserved or not. In patients with preserved systolic function, measurement of transmitral flow in combination with mitral annular tissue Doppler velocities provide the most efficient means of assessing LV diastolic function and filling pressures. However, in case of global systolic dysfunction and accompanying diastolic dysfunction, the principal aim of Doppler echocardiography is to estimate filling pressure, which has known therapeutic and prognostic implications [13]. In these patients, diastolic Doppler parameters are principally used to determine LV filling pressures [13]. Finally, when the regional assessment of the diastolic function is the target, TDI presently represents the best echocardiographic approach.

### 2. Systolic function

Initially described in studies including contrast or radionuclide ventriculography, global and regional left ventricular function are optimally assessed with echocardiographic technique in both resting and stress condition. With the ultrasound beam directed perpendicularly to the myocardium, wall motion and thickening can easily be detected on the imaging plane. Several imaging windows, including parasternal, apical, and subcostal views, are used to visualize all ventricular walls. More recently, three dimensional (3D) echocardiographic images are generated by integration of information from multiple 2D imaging planes. In addition, the introduction of harmonic imaging and the use of echocardiographic contrast media for improved endocardial border definition have significantly improved image quality, thereby enhancing the accuracy of assessment of left ventricular function particularly in patients with poor acoustic windows. Estimation of the LVEF by 2D echocardiography can be done either qualitatively by visual inspection of global and regional function or quantitatively, using geometric assumptions regarding the shape of the LV cavity.

Global and regional LV systolic function can both be qualitatively and quantitatively assessed with echocardiography.

Global function is qualitatively described as normal, depressed, or hyperdynamic. Although a comprehensive quantitative assessment of LV end-systolic and end-diastolic data would be ideal, such an approach is currently impractical for routine evaluation. Several quantitative approaches have been described, with Simpson's rule (disk-area method) being the most accurate for LV volume measurement and for computation of derived values such as LV ejection fraction [14]. Perhaps this assessment may be obtained in the future with further development of 3D techniques and improved automated edge-detection algorithms.

The regional assessment of LV systolic function is assessed by dividing the entire left ventricle into segments. The most popular models recommend the use of 16 or 17 segments [15,16]. A score is then assigned to each segment: (1 = normal, 2 = hypokinesis, 3 = akinesis, 4 = dyskinesis) and a semiquantitative wall motion score index is derived as the ratio between the sum of scores assigned to each segment and the number of visualized segments.

Future developments for the assessment of ventricular function with echocardiography include the use of *3D technique *and *myocardial strain imaging*.

3D echocardiography offers the theoretical advantage to provide incremental benefit over 2D techniques in terms of accuracy and reproducibility and appears to produce estimation of LV ejection fraction similar to radionuclide ventriculography and cardiovascular magnetic resonance [17-21].

The term "strain" refers to an object's fractional or percentage change from its original dimension [22,23], thus reflecting the deformation of a structure. When applied to the myocardium, strain directly describes the contraction/relaxation pattern and can be calculated in several dimensions [23]. Strain rate represents the rate of this deformation [22]. Strain rate imaging involves mathematical subtraction of the whole heart or translational motion from regional thickening velocity using a transmural data set from color-coded tissue Doppler echo [24,25]. This quantification of the spatial distribution of intramural velocities across the myocardium has improved the ability of tissue Doppler echo to reflect directional and incremental alterations in regional and global left ventricular contractility in patients with cardiovascular disease [24-27]. Though very promising for assessing several aspect of myocardial function, strain and strain rate imaging presently are of limited applicability in clinical practice due to several limitations [23], such as complex methodology, technical challenges of image acquisition, susceptibility to artefact and lack of standardization.

## Conclusion

The cause of CHF is still unknown, with the impression, based on morphopathologic data, that its pathogenesis consists in a primary metabolic error of myocytes which lost their ability to relax in the contraction cycle. At ultrastructure these elements are in contraction and this finding may indicate the inability to remove Ca^+2 ^from troponin-tropomyosine complex. The metabolic error could stimulate an abortive hypertrophy with elongation of myocytes by new sarcomeres with normal length without tridimensional myofibrillar iperplasia [1,4].

## Competing interests

The author(s) declare that they have no competing interests.

## Authors' contributions

GB conceived and drafted the manuscript. RG and LC critically revised and helped to draft the manuscript. All authors read and approved the final manuscript.
